# Metagenomics in the Interplay Among Oral and Gut Dysbiosis

**DOI:** 10.3390/metabo16070502

**Published:** 2026-07-16

**Authors:** Morena Munzone, Giorgia Maria Marmo, Alessandro Polizzi, Elena Jovanova, Angela Angjelova, Saturnino Marco Lupi, Gaetano Isola

**Affiliations:** 1 Unit of Periodontology, Department of General Surgery and Surgical-Medical Specialties, School of Dentistry, University of Catania, 95123 Catania, Italy; morenamunzone@gmail.com (M.M.); giorgiamarmo284@gmail.com (G.M.M.); elenajovanova3@gmail.com (E.J.); angela.angjelova@students.stomfak.ukim.mk (A.A.); 2 International Research Center on Periodontal and Systemic Health “PerioHealth”, University of Catania, 95123 Catania, Italy; 3 Department of Clinical Surgical, Diagnostic and Paediatric Sciences, School of Dentistry, University of Pavia, 27100 Pavia, Italy; saturninomarco.lupi@unipv.it

**Keywords:** metagenomics, spatial metagenomics, periodontitis, oral–gut axis, dysbiosis, spatial mapping, bulk sequencing, oral biofilm, microbiome

## Abstract

Periodontitis is a chronic inflammatory disease increasingly recognized as a manifestation of complex microbial dysbiosis extending beyond the oral cavity. Recent advances in spatial metagenomics provide unprecedented resolution to investigate microbial community structure, function, and localization within periodontal niches and along the oral–gut axis. This review aims to explore how spatially resolved metagenomic approaches refine our understanding of the ecological and functional shifts in bacterial populations associated with periodontitis and their systemic implications. By integrating spatial mapping with shotgun metagenomics, we highlight distinct microenvironmental signatures within periodontal pockets, characterized by anaerobic pathobionts, metabolic reprogramming, and localized inflammatory gradients. Furthermore, we examine evidence supporting bidirectional interactions between oral and gut microbiota, suggesting that oral-derived taxa may contribute to gut dysbiosis through translocation and ecological disruption. From a basic science perspective, spatial metagenomics reveals niche-specific microbial functions and interspecies interactions that are not captured by bulk sequencing. Clinically, these insights open avenues for precision diagnostics and targeted therapeutics, including microbiome modulation strategies tailored to spatial microbial organization. Overall, this work underscores the importance of spatial context in metagenomic analyses and advances the conceptual framework linking periodontal disease to systemic microbial dysbiosis.

## 1. Introduction

The term “metagenomics” was introduced in the late 1990s and refers to the study of the collective genetic material recovered directly from environmental or clinical samples without the need for laboratory cultivation of microbial species [1,2]. This approach has revolutionized our understanding of the human microbiome by overcoming the inherent limitations of conventional genomics. Classical microbiology, which relies on microbial isolation and culture, has long been constrained by the difficulty—and, in some cases, the impossibility—of reproducing the environmental conditions required for the growth of many microorganisms in vitro. Consequently, rare or previously uncharacterized microorganisms remained undetected for decades [1,3,4]. This “uncultured majority”, also referred to as “microbial dark matter,” is estimated to account for approximately 98–99% of microorganisms inhabiting the human body, further highlighting the transformative impact of metagenomics on the characterization of microbial communities [5,6,7]. Unlike conventional genomic approaches, which primarily provide taxonomic identification of microbial species, shotgun metagenomics enables comprehensive analysis of the collective microbial genome while simultaneously revealing the functional and metabolic potential of microbial communities through the reconstruction of metabolic pathways and gene functions [7,8].

The metagenomic workflow begins with sample collection from specific biological niches, followed by extraction of total microbial DNA. The extracted DNA is subsequently fragmented and ligated with sequencing adapters to generate a metagenomic library. High-throughput sequencing is then performed using either 16S ribosomal RNA (16S rRNA) gene sequencing or whole-genome shotgun (WGS) metagenomic sequencing. Raw sequencing reads undergo quality control, filtering, and assembly into longer contiguous sequences, enabling both taxonomic and functional annotation. Finally, bioinformatic and statistical analyses are performed to identify microbial signatures and their potential clinical associations [5,9,10,11].

The 16S rRNA gene sequencing approach is based on amplification and sequencing of the bacterial and archaeal 16S ribosomal RNA gene, which contains highly conserved regions interspersed with hypervariable regions that enable taxonomic discrimination among microorganisms. (Application of metagenomics in understanding oral health and disease, Autophagy mediates the impact of Porphyromonas gingivalis on short-chain fatty acids metabolism in periodontitis-induced gut dysbiosis).

In contrast, shotgun metagenomics (Whole Metagenome Shotgun sequencing, WMS) involves random fragmentation and sequencing of genomic DNA, followed by computational reassembly or mapping to reference database. Compared with 16S rRNA sequencing, shotgun metagenomics provides substantially higher taxonomic resolution, allowing identification at both the species and strain levels, while simultaneously characterizing the functional repertoire of microbial communities. This approach enables the identification of genes associated with antimicrobial resistance, virulence factors, metabolic pathways, and other functional traits that cannot be inferred from 16S rRNA profiling alone [5,10,11]. Advances in bioinformatics and decreasing sequencing costs have further expanded its applicability, enabling the study of microorganisms lacking 16S rRNA genes [12]. Within this field, a distinction is made between traditional shotgun metagenomics and emerging spatial metagenomic approaches. Unlike 16S rRNA sequencing, this approach is not restricted to specific taxa and allows high-resolution analysis at species and strain levels, as well as functional pathway characterization. However, it requires sample homogenization, which disrupts spatial organization and microscale interactions [2,12,13,14,15,16]. To better understand the structural organization and functional properties of individual microbial communities, a spatial approach has been proposed in ecology, in which different dimensions are considered. In this context, spatial metagenomics has also been applied to the study of gut, oral, and skin microbiomes. Understanding host–microbiome interactions through emerging technologies enables the exploitation of microbial communities for the diagnosis and treatment of both local and systemic diseases [17].

To this end, novel spatial metagenomics techniques have been developed. The most recent evolution of metagenomic technologies is represented by multidimensional metagenomics, which extends beyond conventional taxonomic profiling. The first dimension encompasses microbial taxonomic composition and relative abundance, whereas the second captures intra-strain genetic variability. The third and fourth dimensions integrate metatranscriptomic analyses and spatiotemporal dynamics, respectively, thereby providing a comprehensive understanding of microbial community structure, function, and ecological interactions within their native environments [18]. These technologies enable in situ mapping of microbial community structure and metabolic potential, providing insights into interspecies interactions and niche-specific functions that are not detectable using conventional approaches. The applications of spatial metagenomics are extensive and include functional and taxonomic mapping of microbiomes, elucidation of pathogenic mechanisms and dysbiosis, identification of novel microorganisms [19,20], monitoring of microbial transmission across individuals and body sites, and discovery of new therapeutic targets and diagnostic biomarkers [19,21]. Much of the current understanding of the oral microbiome, its spatial organization, and its systemic implications has been enabled by metagenomic approaches.

The oral cavity represents a highly heterogeneous ecosystem and one of the most complex microbial habitats after the gut microbiota. The collective genome of all microorganisms inhabiting the oral cavity is referred to as the “oral microbiome”. The term “microbiome” was originally coined by Joshua Lederberg, who laid the theoretical and technological foundations for the Human Genome Project, and is generally defined as the ensemble of pathogenic, symbiotic, and commensal microorganisms that colonize the human body [15,22]. To date, the oral microbiome is known to comprise more than 700 bacterial species (including *Actinobacteria*, *Bacteroidetes*, *Firmicutes*, *Fusobacteria*, *Proteobacteria*, *Saccharibacteria*, and *Spirochaetes*), as well as viruses, fungi, protozoa, and archaea in lower abundance [16,23]. These microbial communities play a fundamental role in maintaining oral health by contributing to immune system maturation and protecting the host against colonization by exogenous pathogens [24]. Another important microbiome is gut one that represents the largest and most densely populated polymicrobial community in the human body, particularly within the colon. It is dominated by *Firmicutes*, *Bacteroidetes*, *Actinobacteria*, and *Proteobacteria*, alongside viruses, fungi, and archaea. [23,25,26]. Its functions are essential for host homeostasis, including the regulation of energy metabolism and digestion through the production of short-chain fatty acids (SCFAs), the synthesis of essential vitamins, modulation of the immune system, and resistance to pathogen colonization [5,23,27,28,29].

A growing body of evidence supports the existence of a functional relationship between the oral and gut microbiomes, commonly referred to as the oral–gut axis, particularly in the context of oral, intestinal, and systemic diseases. Although oral and intestinal tissues share key features, the degree of similarity between the oral and gut microbiomes remains debated [30]. Being located at the two extremities of the digestive tract, they are anatomically continuous and chemically interconnected through the constant swallowing of saliva, which carries oral microorganisms into the gastrointestinal tract; for this reason, the Human Microbiome Project suggests an overlap of approximately 45% between oral and fecal microbiota in healthy individuals [31,32]. However, other studies, such as those reported by Kunath et al., indicate that under physiological conditions the two microbiomes are distinct, largely due to physicochemical barriers that limit the survival and colonization of oral bacteria in the gut, including gastric acidity, bile salts, and pancreatic secretions, as well as differences in oxygen availability, nutrient composition, and pH between the two environments [33]. Importantly, methodological approaches significantly influence these observations: in fact, studies based on 16S rRNA sequencing generally support a clear distinction between oral and gut microbial communities, whereas shotgun metagenomic analyses often reveal a higher degree of similarity, suggesting substantial translocation of oral taxa to the gut [34].

**Figure 1:**
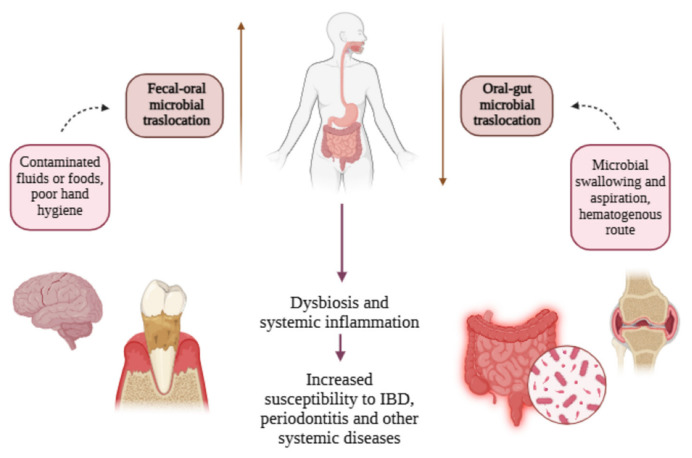
The oral cavity–gut relationship described through microbial translocation phenomena. The figure illustrates the bidirectional microbial translocation between the oral cavity and the intestine, which contributes to the development of dysbiosis and systemic inflammation underlying IBD, periodontitis, and other systemic disorders. Oral-to-gut translocation occurs through continuous saliva swallowing, aspiration, or hematogenous dissemination. Conversely, fecal–oral transmission may occur through the ingestion of contaminated food or fluids, as well as through poor hygiene practices. Created in Created in BioRender. Munzone, M. (2026) https://BioRender.com/outot9u.

Moreover, it remains unclear whether such translocation represents a physiological process or a pathological phenomenon. According to the classical paradigm, colonization of the gut by oral bacteria is a rare event that occurs primarily in the presence of intestinal barrier dysfunction (“leaky gut”), as oral microorganisms are thought to be unable to survive gastric acidity under normal conditions [24,31,35]. In this context, the “multi-hit” hypothesis proposes that effective intestinal colonization by oral bacteria (“oralization”) occurs only when oral and gut dysbiosis coexist alongside impaired barrier function, thereby promoting both local and systemic inflammation [36]. Consistent with this hypothesis, periodontopathogens such as *Porphyromonas gingivalis* and *Fusobacterium nucleatum* have been detected in the feces of patients with IBD (Inflammatory Bowel Disease), colorectal cancer, and liver cirrhosis. More recent evidence indicates that certain oral bacteria, including *P. gingivalis*, can survive gastric conditions and reach the intestine, where they may disrupt barrier integrity and facilitate further colonization by oral pathobionts [10,23]. Meanwhile, current perspectives suggest that continuous microbial transfer from the oral cavity to the gut occurs even under physiological conditions, primarily through swallowing and, to a lesser extent, aspiration. The enteral route is predominant, with an estimated daily ingestion of 10^12^–10^13^ bacteria through saliva (Figure 1) [37,38]. Under conditions of eubiosis, the gut microbiome exerts strong colonization resistance; however, the persistent influx of oral bacteria is sufficiently substantial that oral microbial composition may partially predict gut microbiome structure [33]. Other studies suggest the oral–gut relationship is bidirectional. In fact, the gut can also act as a reservoir for microbial species that subsequently colonize the oral cavity, either transiently or permanently, through mechanisms such as environmental exposure, ingestion of contaminated food or water, poor hygiene practices, and host susceptibility factors, including immune dysfunction (Figure 1) [25]. Human microbiomes and their interactions have sparked a great deal of interest because they are essential for maintaining systemic health, while microbial dysbiosis is implicated in the development of both local and systemic non-communicable diseases [19,39,40]. In this context, the association between the oral microbiome and systemic diseases has been extensively investigated and is largely explained by the bidirectional nature of the oral–gut axis [32].

In the oral cavity, dysbiosis together with host susceptibility represents key determinants in the development of periodontal disease [23,41,42,43]. Periodontitis is defined as a multifactorial chronic inflammatory disease associated with biofilm dysbiosis and mediated by the host response, characterized by the progressive loss of alveolar bone and periodontal attachment, namely the tooth-supporting apparatus [44,45]. In the absence of appropriate treatment, periodontitis results in progressive tooth mobility and eventual tooth loss, with substantial repercussions on oral function, aesthetics, healthcare costs, and patients’ social interactions and quality of life [46]. The global prevalence of periodontitis is estimated to be around 50%, and epidemiological data indicate a rising trend, particularly among younger populations [47].

The conceptual framework of periodontitis pathogenesis has evolved substantially, shifting from a microorganism-centered model based on virulence factors (Ecological Plaque Hypothesis) to a biofilm-centered perspective emphasizing spatial organization and microbial interactions. Currently, the Polymicrobial Synergy and Dysbiosis (PSD) model is widely accepted, proposing that periodontal disease arises from a dysbiotic microbial community that elicits a dysregulated and destructive host immune response, driven by altered cell–cell interactions. Furthermore, the magnitude of the host response—including epithelial inflammation, immune evasion, and immunomodulatory mechanisms—is closely linked to the spatial organization of the biofilm and its proximity to host tissues, ultimately leading to progressive destruction of periodontal connective tissue and alveolar bone [19,23,43].

Periodontitis has been identified as a risk factor for several extraoral diseases, including cardiovascular disease, type 2 diabetes mellitus, rheumatoid arthritis, and neurodegenerative disorders [9,22,27,48,49]. Within the oral–gut axis framework, increased prevalence and severity of periodontitis have been reported in patients with IBD, particularly in those with inadequate oral hygiene [37]. Additionally, gastrointestinal diseases are frequently associated with oral manifestations, such as ulcers, aphthous lesions, and gingivitis, often linked to oral dysbiosis. Experimental studies further support this connection. In animal models, ingestion of *P. gingivalis* alone has been shown to alter gut microbiome composition, impair intestinal barrier function, and induce systemic inflammation. However, it remains unclear whether oral bacteria act as primary drivers or secondary contributors to gut dysbiosis and associated inflammatory processes, highlighting the need for well-designed clinical studies with larger cohorts and careful control of confounding variables. It has been proposed that periodontitis may induce chronic low-grade systemic inflammation, leading to disruption of intestinal barrier integrity and increased susceptibility to conditions such as IBD, tumorigenesis, and other systemic disorders [33]. Conversely, gastrointestinal diseases may exacerbate periodontal pathology; for example, Crohn’s disease can worsen oral dysbiosis through systemic immune dysregulation and reduced tolerance to periodontal bacteria [50,51].

Current knowledge regarding the oral–gut axis derives from heterogeneous sources of evidence, including in vitro experiments, animal models, observational human studies, and a limited number of clinical intervention studies. While experimental models have substantially contributed to elucidating the biological mechanisms underlying microbial translocation and host–microbiome interactions, most human evidence remains observational. Consequently, associations identified through metagenomic approaches should not be interpreted as evidence of causality, and further longitudinal and interventional studies are required to validate these findings.

In this context, the present review aims to critically evaluate recent advances in spatial metagenomics applied to the study of the oral–gut axis in periodontitis, highlighting the interplay between oral and intestinal microbiota in the pathogenesis of local and systemic diseases, and discussing the advantages, limitations, and future perspectives of emerging spatial metagenomic technologies.

## 2. Oral and Gut Dysbiosis During Periodontitis

### 2.1. Oral Dysbiosis During Periodontitis

In physiological conditions, a state of equilibrium exists between the host and the microbiota; when this balance is disrupted, a condition known as dysbiosis is established [52]. The disruption of microbial ecological balance ensues with the predominance of pathogenic bacteria or its virulence over commensal species [52,53]. Many oral diseases, including periodontitis, are associated with alterations of the oral microbiota, influencing both host metabolism and immune responses [54]. In particular, in periodontitis, microbial colonization is facilitated by the complex anatomical and structural organization of periodontal tissues, which provides a favourable environment for the dysbiosis of the subgingival microbiome with the establishment and persistence of pathogenic microbial communities by instigating tissue-destructive inflammation [52,54].

**Figure 2:**
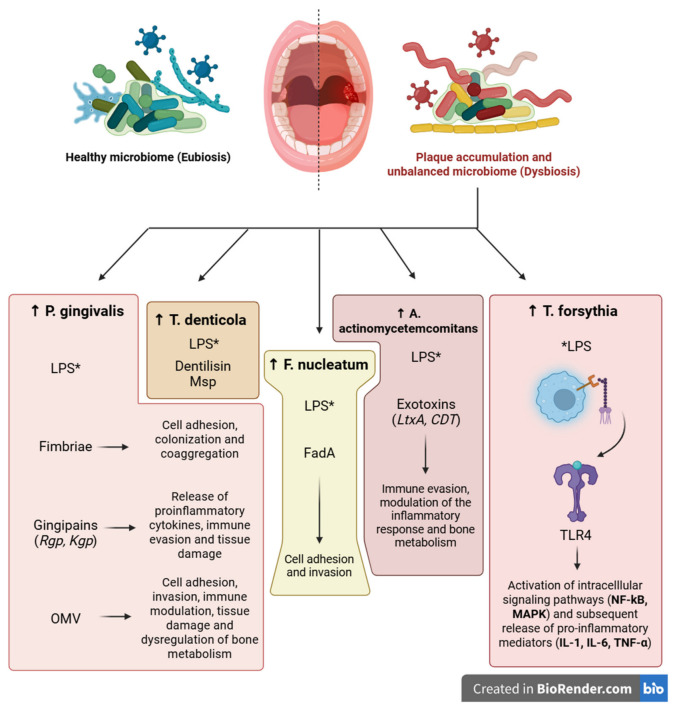
Microbial pathways in periodontitis. Periodontitis is characterized by the development of oral dysbiosis, marked by the enrichment of key periodontal pathogens, including *P. gingivalis*, *T. denticola*, *F. nucleatum*, *A. actinomycetemcomitans*, and *T. forsythia*. Through distinct virulence factors, these microorganisms promote host cell adhesion and invasion, modulate the host immune response, and activate pro-inflammatory signaling pathways, ultimately contributing to periodontal tissue destruction and dysregulation of bone metabolism. Major virulence factors include lipopolysaccharide (LPS*), fimbriae, the adhesin FadA, cysteine proteases (gingipains Rgp and Kgp), the exotoxins LtxA and CDT, dentilisin, Major Sheath Protein (Msp) and outer membrane vesicles (OMVs). Created in BioRender. Munzone, M. (2026) https://BioRender.com/vwzqix7.

Oral microbial dysbiosis is triggered by *P. gingivalis*, which disrupts the symbiotic balance and promotes the proliferation of other pathogens, such as *Tannerella forsythia* and spirochetes, thereby playing a crucial role in the development of periodontal disease [52,54]. *P. gingivalis* induces a strong, tissue-destructive host immune response and the pathogenic mechanisms that ensure its replication, survival within the host, and evasion of the immune system are due to the presence of several factors like fimbriae, lipopolysaccharide (LPS), outer membrane vesicles (OMV), and gingipains [30,55] (Table 1, Figure 2).

Fimbriae are responsible for bacterial adhesion, colonization of host cells, and aggregation with other bacterial species, such as *Treponema denticola*, facilitating its invasion; LPS activates macrophages through Toll-like receptors (especially TLR2 and TLR4), which, together with fimbriae and outer membrane vesicles, cause a strong inflammatory response [54,55]. Inflammation is also sustained by gingipains, which promote the release of pro-inflammatory cytokines, including interleukin-1β (IL-1β), interleukin-6 (IL-6), and tumor necrosis factor-α (TNF-α). Furthermore, arginine-specific gingipains (Rgp) and lysine-specific gingipains (Kgp) inhibit the local immune response and facilitate periodontal tissue damage [30,52,54,56] (Table 1, Figure 2).

*P. gingivalis*, together with *T. forsythia* and *T. denticola*, are classified, according to Socransky’s classification, as a member of the so-called “red complex” [52]. These three bacteria colonize the anaerobic environment of periodontal pockets, contributing to the destruction of periodontal tissues, and form a structured multispecies biofilm that protects them from host immune responses [30,54]. Moreover, studies have demonstrated that their co-presence promotes the growth and virulence of each species [30,54].

Crucially, the pathogenicity of these individual species cannot be fully understood in isolation, as their survival and virulence are tightly regulated by complex inter-species communication networks known as quorum sensing (QS) [9,11]. Subgingival biofilms function as highly coordinated polymicrobial communities rather than mere aggregations of single species organized in well-defined microbial complexes [57]. In this environment, signaling molecules such as Autoinducer-2 (AI-2) act as a universal language that facilitates cross-talk between early colonizers and late pathobionts [58]. This chemical signaling directly regulates the transcription of virulence factors, metabolic cooperation (cross-feeding), and extracellular matrix production across the entire community. For instance, while *F. nucleatum* physically bridges different bacterial layers through co-aggregation proteins [30,54], QS mechanisms simultaneously synchronize the metabolic activity of anaerobic consortia, shifting the local microenvironment toward extreme hypoxia and high hydrogen sulfide (H_2_S) production [53,59]. Consequently, the transition from oral eubiosis to periodontal disease is an emergent property of these synchronized microbially driven networks, where a ‘keystone pathogen’ like *P. gingivalis* can orchestrate the remodeling of a benign microbiota into an aggressive, tissue-destructive disbiotic entity [23,43,49].

**Table 1:** Virulence factors of periodontal bacteria and their effects on periodontal tissue.

Periodontal Bacteria	Virulence Factors	Effects
*P. gingivalis*	Fimbriae [30,55]	Bacterial adhesion, colonization of host cells, and aggregation with other bacterial species [54,55]
*P. gingivalis*	LPS [30,55]	Macrophages’ activation [54,55]
*P. gingivalis*	OMV [30,55]	Inflammation [54,55]
*P. gingivalis*	Gingipains [30,55]	Release of pro-inflammatory cytokines (IL-1β; IL-6 and TNF-α) [30,52,54,56]
*P. gingivalis*	Rgp and Kgp [30,52,54,56]	Inhibition of local immune response and periodontal tissue damage [30,52,54,56]
*T. forsythia*	LPS [54]	Release of pro-inflammatory cytokines (IL-1; IL-6 and TNF-α) [54]
*T. denticola*	LPS [60]	Activation of the host immune response [60]
*T. denticola*	Dentilisin [60]	Host cell invasion, activation of complement proteins [60]
*T. denticola*	Msp [60]	Tissue damage [60]
*A. actinomycetemcomitans*	LtxA [52]	Impairment of anti-inflammatory functions of leukocytes [52,54]
*A. actinomycetemcomitans*	CDT [52]	Impairment of anti-inflammatory functions of leukocytes [52,54]
*A. actinomycetemcomitans*	Metabolic products [52,54]	Impairment of anti-inflammatory functions of leukocytes [52,54]
*F. nucleatum*	LPS [30,54]	Activation of the host immune response through the release of pro-inflammatory mediators [30,54]
*F. nucleatum*	FadA [30,54]	Host cell adhesion and invasion, coaggregation and activation of intracellular signaling pathways [30,54]

Similarly to *P. gingivalis*, *T. forsythia*, through LPS expressed on its cell wall, modulates the inflammatory response by inducing the secretion of pro-inflammatory cytokines such as interleukin-1 (IL-1), IL-6 and TNF-α [54]. Furthermore, *T. denticola* contributes to the degradation of the extracellular matrix (ECM) and serum proteins through the activity of dentilisin and exerts cytotoxic effects via the Major Sheath Protein (Msp) [60] (Table 1, Figure 2).

*Aggregatibacter actinomycetemcomitans* is a Gram-negative facultative anaerobic bacillus, which is primarily detected in the subgingival biofilms of young individuals affected by periodontitis [52]. It is characterized by the expression of two exotoxins: a leukotoxin (LtxA) and a cytolethal distending toxin (CDT) [52]. LtxA plays a key role in aggressive periodontitis by impairing the anti-inflammatory functions of leukocytes, whereas CDT contributes to disease pathogenesis by modulating inflammatory responses and bone metabolism. Furthermore, the metabolic products of *A. actinomycetemcomitans* stimulate the release of pro-inflammatory cytokines, including TNF-α and IL-1β [52,54].

### 2.2. Microenvironmental Signatures Within Periodontal Pockets

Subgingival bacterial colonization of the tooth surface leads to the formation of the periodontal pocket, defined as the pathological deepening of the gingival sulcus, characterized by apical migration of the junctional epithelium relative to the gingival margin, occurring because of clinical attachment loss [61]. Within the pocket, disruption of the junctional epithelium is promoted both by bacteria such as *P. gingivalis*, which proteolytically degrade epithelial cell–cell junctions through the action of gingipains, and by the intense inflammatory response, which induces neutrophil transmigration across the epithelium, resulting in the widening of intercellular spaces [61,62]. The periodontal pocket plays a key role in the progression of periodontitis, as the impairment of the junctional epithelium facilitates microbial invasion into the underlying connective tissue, thereby establishing a self-perpetuating cycle of tissue destruction [61,62].

In recent years, increasing attention has been directed toward the metabolic activities of the biofilm and their potential impact on periodontitis [53] (Table 2). Several studies have demonstrated that the microbiota within the periodontal pocket is predominantly composed of Gram-negative, strictly anaerobic species, whose main metabolic pathway is protein fermentation [53,59]. Accordingly, several genera, such as *Porphyromonas*, are asaccharolytic and degrade peptides and amino acids into short-chain fatty acids (SCFAs), NH_3_, sulfur-containing compounds, and amines, which at high concentrations may exert detrimental effects on the host [53]. SCFAs include formic acid, acetic acid, propionic acid, butyric acid, and valeric acid. Acetate is abundantly produced by *F. nucleatum* and *P. gingivalis*; moreover butyric acid and propionic acid are found at increased levels in the subgingival plaque and gingival crevicular fluid (GCF) of patients with periodontitis [53]. Collectively, these acids contribute to immunosuppression. Indeed, they significantly inhibit the proliferation of T and B lymphocytes, as well as the production of several key cytokines by T lymphocytes, including IL-2, IL-4, IL-5, IL-6, and IL-10 [63].

Bacterial fermentation by several species, including *Porphyromonas* spp., *Fusobacterium* spp., *Alloprevotella tannerae*, *Parvimonas micra*, *T. denticola*, *V. parvula*, and *S. anginosus*, leads to the production of hydrogen sulfide (H_2_S), resulting in elevated concentrations within periodontal pockets [53,64]. In this microenvironment, H_2_S exerts pro-inflammatory effects by activating the NLRP3 inflammasome in macrophages, leading to the secretion of IL-1β and IL-18. It also stimulates the production of IL-6 and IL-8 in fibroblasts and epithelial cells, induces apoptosis in gingival fibroblasts, periodontal ligament cells, and lymphocytes, and interferes with collagen synthesis, thereby impairing tissue repair processes [53,64].

Other studies have demonstrated that oxygen tension may also influence the extent of tissue damage. The literature indicates that increased periodontal pocket depth is associated with greater hypoxia, with oxygen levels reaching approximately 2% [56]. Under these conditions, *Prevotella intermedia*, a member of the orange complex, and *P. gingivalis*, are able to maintain and further potentiate periodontal inflammation through activation of NF-κB and upregulation of pro-inflammatory genes such as *TNF-α*, *IL-1β*, and *IL-8*. These mechanisms contribute to connective tissue destruction and increased pocket depth [56].

In recent years, a novel approach for the management of deep periodontal pockets associated with intrabony defects has been introduced, namely Minimally Invasive Non-Surgical Therapy (MINST). This procedure is performed under local anesthesia and involves full-mouth periodontal debridement in a single session using ultrasonic instruments equipped with slim tips, mini-curettes, and magnification devices. MINST may represent a valid alternative to conventional quadrant-based non-surgical periodontal therapy, as it appears to be more effective in reducing inflammation, treatment and healing times, and patient morbidity [65].

### 2.3. Gut Dysbiosis During Periodontitis

Periodontal bacteria, after inducing oral dysbiosis, can disseminate and colonize the gastrointestinal tract via both the circulatory system and ingestion [29]. The spread of these pathogens disrupts the gut microbiota, establishing a dysbiotic state that promotes the onset of several systemic conditions, including type 2 diabetes, IBD, and non-alcoholic fatty liver disease (NAFLD). Therefore, a dynamic interplay exists between the oral and intestinal environments, which may significantly influence systemic health [27,29,66].

Observational human evidence in the study by Lourenço et al., conducted on fecal samples from patients with periodontitis, demonstrated that these individuals exhibit alterations in the gut microbiome, characterized by a reduction in *Bacteroidetes* and an increase in *Proteobacteria*, *Verrucomicrobia*, and *Euryarchaeota*. *Furthermore*, the study identified the presence of periodontal bacteria, including *Fusobacterium*, *Tannerella*, and particularly *Prevotella* and *Porphyromonas*, not only in patients with periodontitis and gingivitis but also in periodontally healthy individuals [67].

Experimental evidence derived primarily from murine models suggests that oral pathobionts, including *P. gingivalis*, are capable of translocating to the gastrointestinal tract, where they activate inflammasome signaling in lamina propria macrophages. Although these findings provide important mechanistic insights, direct evidence demonstrating the same sequence of events in humans remains limited. [27]. Upon reaching the intestine, these cells are reactivated by translocated oral pathobionts, thereby amplifying the intestinal inflammatory response and contributing to colitis.

Animal studies further indicate that intestinal colonization by oral pathogens may promote Th17-cell differentiation within Peyer’s patches, thereby amplifying intestinal inflammation. Whether this mechanism plays an equivalent role in human periodontitis-associated gut dysbiosis has not yet been conclusively demonstrated [27]. Experimental models of periodontitis suggest that pre-existing intestinal dysbiosis may aggravate alveolar bone loss, supporting the concept of bidirectional communication between the gut and periodontal tissues. However, confirmation in longitudinal human studies is still lacking. Finally, recent experimental and genetic evidence suggests that members of the Enterobacteriaceae family may contribute to periodontal inflammation by acting synergistically with classical periodontal pathogens. Nevertheless, the clinical relevance of this interaction remains to be confirmed in prospective human studies [68]. Therefore, a bidirectional crosstalk exists between the oral and intestinal environments, establishing a dynamic inflammatory cycle in which inflammation in one site fuels inflammation in the other [27,50,51].

Gut dysbiosis impairs intestinal barrier function and can ultimately lead to its disruption [29]. Indeed, in a murine model Nakajima et al. demonstrated that experimental oral inoculation of mice with *P. gingivalis* compromises intestinal barrier integrity, resulting in subsequent systemic endotoxemia. Specifically, *P. gingivalis* alters the intestinal epithelium by reducing the expression of tight junction proteins such as Tjp1 (ZO-1) and occludin (Ocln) [69]. Moreover, in obese murine models of type 2 diabetes, it induces enterohepatic metabolic disturbances that lead to increased blood glucose levels [69]. Although these findings strongly support a mechanistic link between oral dysbiosis and intestinal dysfunction, equivalent causal evidence in humans is currently unavailable.

At the intestinal level, *P. gingivalis* also plays a key role in the pathogenesis of colorectal cancer (CRC); it triggers a proinflammatory response, leading to tissue alterations that promote intestinal dysregulation. *F. nucleatum* is rarely detected in the gut of healthy individuals, whereas it is highly prevalent in patients with CRC, where it contributes to enhanced cancer cell proliferation, the establishment of a tumor-promoting immune microenvironment through miRNA-mediated activation of TLR2/TLR4 signaling, and the evasion of immune checkpoint mechanisms [29,70]. Accumulating evidence suggests that oral pathogens, particularly *P. gingivalis* and *Fusobacterium nucleatum*, may contribute to colorectal carcinogenesis by promoting chronic inflammation, immune modulation, and epithelial dysfunction. However, most mechanistic evidence derives from experimental studies, whereas human data are predominantly observational and therefore do not establish a direct causal relationship.

*Helicobacter pylori* has also been detected in periodontal pockets and is regarded as a potential source contributing to reinfection in gastroesophageal infections. Furthermore, it may influence the prognosis of patients with gastric, colorectal, and colon cancers [29].

However, it remains unclear whether oral bacteria act as primary drivers or secondary contributors to gut dysbiosis and associated inflammatory processes, highlighting the need for well-designed clinical studies with larger cohorts and careful control of confounding variables [29]. Overall, current evidence supporting the oral–gut axis is characterized by marked heterogeneity in study design. Mechanistic understanding largely derives from experimental animal models, whereas human evidence is predominantly observational and based on metagenomic association studies. Consequently, although the biological plausibility of oral bacterial translocation is strong, causality between oral dysbiosis and gastrointestinal diseases has not yet been conclusively established. Future longitudinal cohort studies, intervention trials, and spatial metagenomic investigations will be essential to clarify temporal relationships and validate these mechanisms in humans.

### 2.4. The Link Between Periodontal Bacteria and Their Systemic Implications

The oral–gut axis represents a critical conduit through which oral dysbiosis may promote chronic inflammation and the development of systemic diseases, including inflammatory bowel disease, neurological disorders, diabetes, cardiovascular diseases, and rheumatoid arthritis [71]. Metabolomic profiling has enabled the investigation of systemic biochemical alterations occurring along the oral–intestinal axis in patients with periodontitis. Studies conducted on fecal samples have revealed reduced activity of commensal gut bacteria responsible for producing SCFAs, which are known for their anti-inflammatory effects, alongside increased levels of other metabolites, including succinate, trimethylamine, methylamine, formate, and 3-hydroxyphenylacetate [27,72,73]. These conditions are associated with an elevated risk of systemic inflammation and are commonly observed in disorders such as IBD, metabolic syndrome, and cardiovascular diseases [27,72,73].

In addition, patients with periodontitis exhibit elevated urinary levels of claudin-2, indicative of increased intestinal permeability, immune dysregulation, and systemic inflammation, as well as reduced salivary lactoferrin levels, a condition associated with exacerbated inflammatory responses [71]. Finally, studies have demonstrated that *P. gingivalis*, by inducing and exploiting intestinal barrier disruption and neuroinflammation, contributes to cognitive impairment and exacerbates Parkinson’s disease [74].

## 3. Role of Spatial Metagenomics as a Source of Early Diagnosis of Oral Dysbiosis

The traditional method for studying microorganisms is based on bacterial culture on specific culture media under laboratory conditions. Over time, this technique has proven to be limited due to the inability to isolate and culture certain microbial species. To overcome this issue, the development of modern technologies has led to a significant evolution in microbiome research approaches. Indeed, recent decades have witnessed the emergence of the so-called “omics” sciences, aimed at investigating entire classes of biological molecules to further elucidate microbiome–host interactions [10].

**Figure 3:**
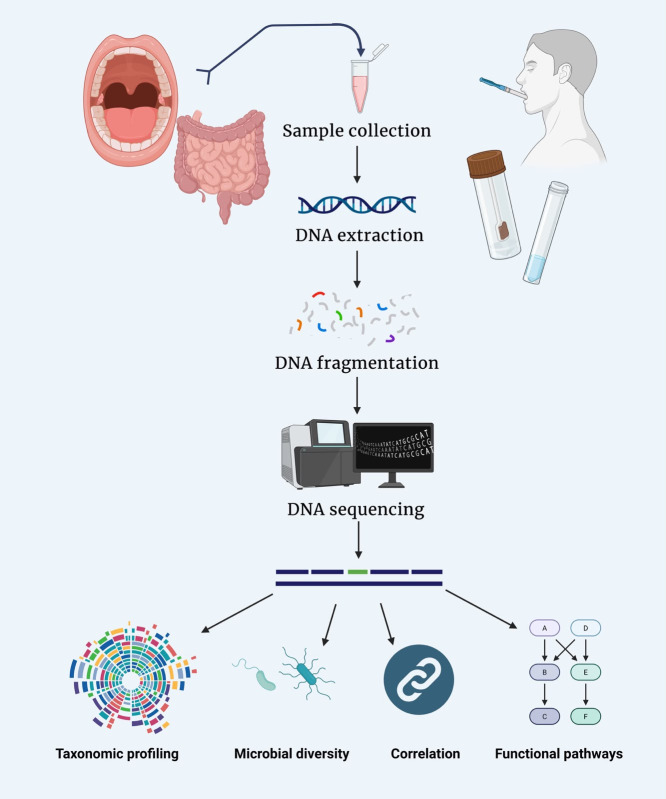
Operational workflow of metagenomics. The process begins with the collection and preservation of biological material obtained from specific ecological niches. Subsequently, total DNA is extracted and purified in order to isolate the microbial genomic component. This is followed by the preparation of the metagenomic library through DNA fragmentation into 300–500 bp fragments and the addition of adapters and barcodes. The resulting material is then sequenced and computationally processed to assess sequence quality and assemble larger genomic fragments. The obtained data can subsequently be used for taxonomic assignment, microbial diversity studies, correlation analyses, and investigation of functional pathways. Created in BioRender. Munzone, M. (2026) https://BioRender.com/6dx7gz4.

Metagenomics, in particular, represents one of the major branches of omics sciences and consists of the application of gene sequencing methods for the analysis of the complete genomic content of multiple microbial species within a sample [75,76,77]. The workflow required to obtain analyzable data involves a series of fundamental steps, illustrated in Figure 3.

By exploiting modern next-generation sequencing (NGS) technologies, metagenomic approaches have substantially expanded current knowledge regarding both the taxonomic composition and functional characteristics of the oral biofilm, particularly during the transition from health to disease. Owing to its cultivation-independent nature, metagenomics has reduced the proportion of unknown species within the oral microbiome from approximately 50% in the past to nearly 30% today [78] (Table 3). The metabolic and functional characterization of these newly identified taxa has revealed novel and intriguing insights into the pathogenesis of periodontitis. In fact, newly discovered species and previously uncultivable phylotypes (therefore not included among the traditionally recognized periodontopathogens) have demonstrated potentially pathogenic behavior, exhibiting metabolic activities and virulence factors that contribute to the onset and progression of periodontal disease [9,79]. Furthermore, metagenomics also represents a valuable tool for the early identification of signs of oral dysbiosis [10].

Unlike previous 16S rRNA-based techniques, shotgun metagenomics provides additional information regarding the genomic potential of the analyzed microbial populations [78] (Table 3). This approach has been applied, for example, to investigate the microbiome of diabetic patients in order to identify functional signatures associated with susceptibility to periodontitis. Evidence has shown that diabetic patients exhibit reduced microbial diversity and subgingival microbiome dysbiosis compared with healthy individuals, thereby supporting the hypothesized pathogenic mechanisms underlying the increased susceptibility to periodontitis in diabetes. However, according to Teles et al., these studies are still limited by the relatively small sample size of enrolled patients [78].

Metagenomic sequencing has enabled comparisons between the bacterial and metabolic profiles of healthy individuals and patients with periodontitis, revealing a greater microbial burden and bacterial diversity in periodontal subjects than in healthy controls [75,80] (Table 3). Conversely, a study by Ai et al. compared the subgingival plaque of healthy subjects and patients with mild and moderate periodontitis through shotgun metagenomics and reported a gradual and progressive loss of microbial diversity in periodontitis patients (alpha diversity), together with alterations in microbial composition closely associated with disease status [9,81].

Although shotgun metagenomics has substantially advanced the characterization of periodontal microbial communities, the available evidence should be interpreted with caution. Most studies conducted to date are cross-sectional and include relatively small patient cohorts, limiting statistical power and preventing causal inference. Moreover, considerable methodological heterogeneity—including differences in sampling strategy, DNA extraction protocols, sequencing depth, taxonomic annotation pipelines, and reference databases—reduces comparability across studies and may partially explain the inconsistent findings regarding microbial diversity in periodontitis. Consequently, although shotgun metagenomics consistently identifies dysbiosis-associated microbial signatures, the reproducibility and clinical generalizability of these biomarkers remain to be established through larger, standardized longitudinal studies.

From a metabolic perspective, bacterial species associated with periodontitis exhibit specific metabolic profiles linked to a parasitic-like behavior, involving pathways related to fatty acid metabolism, ferredoxin oxidation, and acetyl-CoA degradation [75,82,83]. In healthy conditions, symbiotic species express genes associated with periodontal tissue protection, including pathways involved in homoserine, aspartate, and glycerol-3-phosphate metabolism, as well as stress-response genes [84]. Metagenomic analysis has proven useful in identifying early signs of oral dysbiosis in sites that will subsequently progress toward periodontitis, even before clinical manifestation of the disease [84] (Table 3). Already at baseline, sites considered “active” display a higher abundance of pathogens (*Porphyromonas*, *Treponema*, *Tannerella*, and *Prevotella*) compared with protective species such as *Streptococcus*. This analysis has been further refined through metatranscriptomic investigations aimed at identifying metabolic processes indicative of dysbiosis, including nutrient transport, biosynthesis of structural components, and cellular and flagellar motility [84]. Omics-based approaches overcome the limitations of traditional clinical markers (such as bleeding on probing or pocket depth) because they detect microbial physiological changes preceding tissue destruction. Sites that appear clinically healthy or stable in diseased individuals may already exhibit molecular signatures characteristic of disease, indicating that dysbiosis is already ongoing at a microscopic level [84].

Recent evidence has exploited shotgun metagenomics to investigate the existence of the oral–gut axis, particularly in the context of interactions between oral, intestinal, and systemic diseases [85]. For instance, in a cross-sectional metagenomic study Hu et al. compared the oral and gut genomes of healthy subjects and patients with Crohn’s disease (CD), revealing ectopic intestinal translocation of oral bacteria, particularly *S. salivarius*. Furthermore, the study supports the hypothesis that such colonization mainly occurs during active phases of the disease, in agreement with previous findings. Nevertheless, the study is not without limitations, mainly related to the small sample size and the inclusion of only an Asian population [85].

In a case–control shotgun metagenomic study by Qin et al. compared the oral and fecal microbiomes of patients with gastric cancer (GC) and chronic gastritis (ChG) using shotgun metagenomics, confirming oral–intestinal transmission of bacterial species belonging to *Streptococcus* and *Lactobacillus* in GC patients. The authors suggested that the environment generated during GC progression (for example, reduced gastric acidity) may create a favorable microenvironment for colonization and proliferation of these bacteria. However, the results cannot be generalized because of several limitations, including the lack of oral status assessment, study design issues, and limitations related to the metagenomic approach employed [86].

An observational case–control study by Nagata et al. conducted a metagenomic study aimed at identifying microbial signatures associated with pancreatic cancer. Their findings demonstrated that several oral bacteria were enriched in the gut microbiome of pancreatic cancer patients compared with healthy controls, including *Streptococcus oralis*, *Streptococcus vestibularis*, *Streptococcus anginosus*, *Veillonella atypica*, and *Veillonella parvula* [87]. The use of this technique has also confirmed the oral–gut translocation of *F. nucleatum*, one of the major periodontopathogens associated not only with periodontitis but also with IBD and colorectal cancer [86].

Collectively, these studies support the hypothesis that oral microorganisms may translocate to the gastrointestinal tract under dysbiotic conditions. However, current evidence remains largely associative. Most investigations rely on metagenomic profiling rather than direct demonstration of microbial migration, making it difficult to distinguish true colonization from transient bacterial passage or shared ecological signatures. In addition, variations in study populations, disease stage, geographic origin, and analytical workflows further complicate direct comparisons and limit external validity.

Despite the remarkable achievements of shotgun metagenomics in microbiome analysis, this technique presents several limitations, including sample homogenization, which inevitably eliminates the spatial component of the biofilm. [88,89]. To overcome this limitation, a novel spatial metagenomic approach has been developed, taking into account the physical structure of the biofilm, which consists of three distinct dimensions: macroscale, mesoscale, and microscale [19,90] (Table 3). Spatial approaches are essential for understanding biofilm function through the analysis of the relative abundance of microbial species on specific host surfaces and the changes associated with biochemical gradients [19,90] (Table 3). Spatial metagenomics has revealed that dental plaque is a complex and non-random biofilm structure characterized by a three-dimensional organization. Microorganisms tend to distribute across body regions and tissues according to a non-random architecture. This organization depends on several factors, including microbial interactions, host-related factors, and the biofilm matrix [19]. Within the oral cavity, microbial communities are structured both spatially and compositionally. Indeed, although oral surfaces are immersed in the same biological fluid (saliva), they are inhabited by remarkably different microbial communities in terms of relative abundance, organization, and biological interactions. This concept is currently recognized in microbiology as the site-specialist hypothesis [91].

**Table 3:** Comparative Advantages of Shotgun Metagenomics and Spatial Metagenomics.

Shotgun Metagenomics	Spatial Metagenomics
Reduction in the proportion of unknown species within the oral microbiome [78]	It had revealed that dental plaque is a complex and non-random biofilm structure characterized by a three-dimensional organization [19,90]
It provides additional information regarding the genomic potential of the analyzed microbial populations [78]	It is essential for understanding biofilm function through the analysis of the relative abundance of microbial species on specific host surfaces and the changes associated with biochemical gradients [19,90]
Comparisons between the bacterial and metabolic profiles of healthy individuals and patients with periodontitis [75,80]	MSSM ^1^ enables the investigation of microbial communities at the microscale through laser microdissection of tissue samples combined with bioinformatic analyses [88]
Identification of early signs of oral dysbiosis in sites that will subsequently progress toward periodontitis, even before clinical manifestation of the disease [84]	MSSM ^1^ integrates the spatial dimension to better understand microbial dynamics within a specific ecological niche [88]
Investigation of the existence of the oral–gut axis, particularly in the context of interactions between oral, intestinal, and systemic diseases [85]	MSSM ^1^ allows quantitative, strain-resolved, and functionally informed profiling directly from the original tissue. Unlike other techniques, it can reconstruct complete genomes directly from micro-samples [88].

^1^ Micro-scale Spatial Metagenomics.

Furthermore, evidence suggests that the oral cavity acts as a reservoir of microorganisms potentially capable of colonizing the gut as opportunistic pathogens [85]. Such colonization may be facilitated by several host-related conditions, including diarrhea, intestinal inflammation, and the use of anti-acid medications such as proton pump inhibitors [85,92,93,94,95].

Similarly to the oral cavity, the gastrointestinal tract (GIT) can also be considered an anatomical system composed of several distinct yet closely interconnected microbial habitats. The presence of predominantly intestinal microbial species within the oral cavity has been documented in the literature, including *Phocaeicola vulgatus*, trace amounts of *Bacteroides uniformis*, and *Anaerostipes hadrus* in patients with CD and oral ulcers, suggesting possible fecal-to-oral translocation and further supporting the bidirectional nature of the oral–gut axis [41,96].

The integration of spatial mapping and high-resolution sequencing transforms metagenomics into a powerful preventive diagnostic tool. Indeed, it enables the identification of preclinical biomarkers and signs of dysbiosis before the onset of clinical symptoms [10]. For example, *Anaerolineaceae bacterium* HOT-439 has emerged as a promising candidate biomarker requiring external validation for periodontitis. Specifically, an increased relative abundance of this microbial species has been detected in periodontitis patients compared with healthy individuals, and its significance is maintained even in patients with type 2 diabetes. Therefore, *Anaerolineaceae bacterium* HOT-439 may potentially serve as a biomarker even in the presence of systemic comorbidities [97].

Before the development of metagenomics, research on the spatial organization of biofilms was limited mainly by the techniques used for data collection. Fluorescence-based imaging techniques (such as fluorescence in situ hybridization, FISH) and amplicon sequencing techniques (such as 16S rRNA sequencing) were unable to capture the complexity of polymicrobial communities fully and could not identify functional characteristics at the microscale level. Consequently, novel spatial metagenomic techniques such as Micro-scale Spatial Metagenomics (MSSM) were introduced [88]. According to Pietroni et al., unlike traditional shotgun metagenomics, MSSM enables the investigation of microbial communities at the microscale through laser microdissection of tissue samples combined with bioinformatic analyses [88]. Traditional shotgun metagenomics, by contrast, provides a broad range of information derived from large DNA samples without offering insights into microscale organization or spatial resolution. MSSM integrates the spatial dimension to better understand microbial dynamics within a specific ecological niche. Furthermore, MSSM allows quantitative, strain-resolved, and functionally informed profiling directly from the original tissue. Unlike other techniques, it can reconstruct complete genomes directly from micro-samples [88] (Table 3).

Despite its remarkable potential, spatial metagenomics remains an emerging technology. Most currently available applications are proof-of-concept studies, and standardized protocols for tissue processing, spatial resolution, bioinformatic integration, and data interpretation are still lacking. Furthermore, the high costs, limited accessibility of specialized instrumentation, and computational complexity currently restrict its adoption to research settings. Therefore, although spatial metagenomics offers unprecedented opportunities to investigate microbial organization within biofilms, further technological refinement and clinical validation are required before its routine implementation in precision dentistry.

While MSSM provides unprecedented insights into the three-dimensional taxonomic architecture and genomic potential of microbial communities within periodontal pockets and the gastrointestinal tract [88], DNA-based spatial sequencing alone cannot capture immediate physiological activities, active transcriptomic shifts, or localized biochemical outputs [9,11,33,96]. To comprehensively decipher the functional dynamics of the oral–gut axis, spatial metagenomics must be systematically integrated with other spatially resolved “omics” approaches, shifting the paradigm from purely genetic potential to active functional states [98]. Spatial metabolomics, utilizing advanced imaging technologies such as Matrix-Assisted Laser Desorption/Ionization Mass Spectrometry Imaging (MALDI-MSI) and Desorption Electrospray Ionization (DESI-MSI), allows for the direct in situ mapping of metabolites at a micron scale without disrupting the delicate microenvironmental gradients of the biofilm matrix [99,100]. By overlaying high-resolution metabolite maps onto spatial metagenomic grids, researchers can precisely correlate the physical biogeography of specific pathobionts (such as the “red complex” triad or *F. nucleatum*) [30,52,54,86] with the localized accumulation of tissue-destructive or immunomodulatory metabolic by-products [53].

This spatial multi-omics integration is crucial for unraveling host–microbe metabolic interactions occurring at the microscopic borders of host tissues [19,23,43]. For instance, combining spatial metagenomics with metabolite mapping can visually trace how anaerobic subgingival consortia ferment peptides into short-chain fatty acids (SCFAs), such as butyric and propionic acid, or generate high localized concentrations of hydrogen sulfide (H_2_S) within specific hypoxic niches of the periodontal pocket [53,59,64]. Simultaneously, spatially resolved transcriptomics can map the corresponding cellular response of the pocket’s junctional epithelium [101], demonstrating how these localized metabolic gradients activate pro-inflammatory cascades (e.g., NF-κB and NLRP3 inflammasome signaling) and promote localized tissue degradation, apoptosis, and leukocyte transmigration [49,53,56,61,63]. Similarly, along the intestinal mucosa, integrated spatial multi-omics can delineate how translocated oral pathobionts alter the localized production of protective mucosal metabolites, directly mapping the physical sites of tight junction disruption (such as ZO-1 and occludin downregulation) and subsequent immune cell activation [27,29,69]. Ultimately, bridging spatial metagenomics with spatially resolved transcriptomic, metaproteomic, and metabolomic datasets allows for the generation of true multi-layered functional maps of host-microbiome interaction zones [98,101], transitioning microbiome research from purely descriptive biogeography to precise, mechanism-driven diagnostics and targeted spatial therapeutics [10,102].

## 4. Current Limitations

Current limitations of metagenomic techniques are numerous and partially compromise both the applicability and reliability of the obtained results.

The first major limitation concerns costs: metagenomics requires a substantially greater financial investment compared with 16S rRNA sequencing, making it prohibitive for large-scale studies [43,103]. Despite the remarkable advances in NGS technologies, the large volume of material subjected to analysis requires prolonged sequencing runs as well as extensive and highly complex computational resources [9,43,103].

In some cases, taxonomic classification may be further complicated by incomplete databases and the so-called “Microbial Dark Matter.” Indeed, several sequences and genes cannot yet be functionally or metabolically characterized because the relevant data are still absent from current databases [4,10,43,104].

From a technical perspective, metagenomics still lacks a universally standardized protocol for sample collection, DNA extraction, sequencing procedures, and data interpretation, thereby limiting comparability among different studies. DNA extraction procedures themselves are also prone to bias, meaning that conclusions derived from these analyses may not always be entirely accurate [7,10,43,105].

The use of saliva as a representative sample of oral biofilm may be advantageous because of its ease of collection; however, it may not accurately reflect the composition of more structured biofilms, such as those found in the subgingival environment [86,105]. Within the analysis of a single sample, despite the high sensitivity of the technique, the most prevalent species tend to account for the vast majority of sequencing data, thereby masking less abundant or rare species [4]. Another important limitation is contamination by host DNA. In the oral cavity, host-derived DNA may account for 50% to 90% of the total genomic component within the sample. This issue becomes even more critical in samples derived from oral niches characterized by a low microbial DNA content [43,88,106]. Furthermore, metagenomic analyses provide information regarding the relative abundance of microbial strains within a sample, making statistical correlations particularly complex [2]. Unlike other omics sciences, metagenomics exclusively investigates the genome and therefore only assesses the functional potential of microbial species, without distinguishing between living, transcriptionally active, or dead microorganisms. For this reason, metagenomic data should be integrated with other omics approaches, such as metatranscriptomics, metaproteomics, and metabolomics, in order to achieve a more comprehensive understanding of the metabolic potential and biochemical activities of microbial communities [9,11,33,96].

Only through systematic integration among the different modern omics sciences will it be possible to achieve a deeper understanding of the biological and molecular mechanisms underlying resident and colonizing microbial communities of the oral cavity. Such knowledge may further elucidate the pathogenic mechanisms involved not only in oral diseases but also in systemic disorders, with significant implications for prevention, early diagnosis, and therapeutic strategies. Collectively, these limitations highlight that current metagenomic approaches should not be considered standalone diagnostic tools. Rather, they are complementary technologies whose clinical value will depend on the development of standardized protocols, harmonized analytical pipelines, and integration with complementary omics datasets and clinical phenotyping. Overall, available evidence strongly supports the existence of biological interactions between the oral and gut microbiomes. However, current mechanistic knowledge relies predominantly on animal models, whereas clinical evidence in humans remains largely observational. Therefore, although spatial metagenomics offers unprecedented opportunities to investigate microbial ecology, its clinical implementation will require standardized methodologies, multicenter validation studies, and prospective investigations capable of establishing causal relationships.

## 5. Future Directions

Non-surgical periodontal therapy (NSPT), using different hand and ultrasonic instrumentations methods, has a significant impact not only on oral health but also on gut microbiota composition, contributing to the restoration of an ecological balance resembling that observed in healthy individuals [72,107,108]. NSPT can be performed either using a quadrant-based approach (Q-SI), involving treatment sessions scheduled at intervals of 1–4 weeks, or through a full-mouth subgingival instrumentation approach (FM-SI), completed within a single treatment phase over a 24–48 h period. The latter approach appears to be more effective in reducing the burden of periodontal pathogens, particularly in deep periodontal pockets [102].

Furthermore, enrolling patients in a Supportive Periodontal Care (SPC) program following active periodontal treatment contributes to the stabilization of the achieved clinical outcomes. SPC consists of individualized interventions specifically tailored to the patient’s periodontal status and risk profile [109].

Studies have shown that NSPT leads to a reduction in oral microbial diversity, accompanied by an improvement in dysbiotic conditions and the establishment of a healthier and more controlled microbial environment. Furthermore, salivary microbiome analyses have revealed a significant decrease in periodontal pathogen-associated genera, including *Porphyromonas*, *Tannerella*, and *Treponema* [72]. Similarly, in peri-implant mucositis (PM), non-surgical peri-implant submarginal instrumentation (NSPI) combined with chlorhexidine (CHX) has been associated with a significant reduction in the levels of *T. denticola* [110].

Given the bidirectional interaction between the oral cavity and the gastrointestinal tract, NSPT also induces favourable changes in the gut microbiota. following treatment, a reduction in the abundance of genera such as Bacteroides has been observed, while the fecal *Firmicutes*/*Bacteroidetes* ratio, which is altered in patients with periodontitis, increases significantly. Moreover, genera such as *Porphyromonas* become undetectable after treatment [72,107]. Furthermore, in cases of severe and rapidly progressing periodontitis, the use of salicylic acid has been reported to exert a prolonged impact on the gut microbiome, leading to an increased abundance of genes associated with antimicrobial resistance [111].

These findings suggest that NSPT exerts beneficial effects not only on oral health but also on gastrointestinal and systemic health by mitigating microbial alterations that may contribute to the development and progression of chronic inflammatory diseases [107].

Spatial metagenomics, through 3D analysis of microbial communities, is opening new avenues for microbiome modulation [112]. Modern technologies may facilitate the reconstruction of microbial ecological niches, enabling innovative therapeutic strategies. Such approaches involve controlled colonization interventions, whereby selected microbial strains are introduced to exploit their ability to occupy and stabilize specific spatial locations within a given environment, such as intestinal crypts [112]. Furthermore, the integration of spatial metagenomics with transcriptomics and metabolomics enables the generation of multilayered maps of host–microbiome interaction zones, providing insights into how the microbiome influences health and disease at the local level. This integrated framework could support the development of personalized therapeutic interventions that account for the unique spatial and functional architecture of an individual’s microbiome [112]. Nevertheless, several challenges must be overcome before these approaches can be translated into routine clinical practice. Future studies should prioritize multicenter validation, longitudinal cohort designs, cost reduction, standardized analytical workflows, and the development of clinically interpretable bioinformatic pipelines. Without these advances, the transition from experimental research to precision medicine will remain limited.

## 6. Conclusions

In conclusion, the evidence reviewed highlights the complex microbial dysbiosis involving the entire oral–gut axis. The available evidence supports the existence of a bidirectional interaction between the oral and gut microbiomes. Experimental animal studies indicate that oral pathogens may translocate to the gastrointestinal tract and contribute to intestinal barrier dysfunction and promote a systemic inflammatory cycle that contributes to the development and progression of diseases such as diabetes mellitus, rheumatoid arthritis, and IBD. However, human evidence remains largely observational, and further longitudinal and interventional studies are required to establish causality.

The adoption of spatial metagenomics represents a significant advancement over conventional sequencing approaches. From a clinical perspective, these innovations pave the way for precision diagnostics by enabling the identification of early biomarkers and dysbiotic signatures before the onset of overt clinical symptoms. Furthermore, evidence demonstrating that NSPT can induce beneficial changes not only within the oral cavity but also in the gut microbiome underscores the importance of an integrated therapeutic approach for improving overall patient health.

Ultimately, the future of both research and clinical practice lies in the systematic integration of spatial metagenomics with other omics technologies, including metatranscriptomics and metabolomics. This comprehensive framework will facilitate the development of personalized therapeutic strategies and targeted microbiome-modulating interventions aimed at stabilizing microbial communities within their specific anatomical niches, thereby significantly enhancing the prevention and management of dysbiosis-associated systemic diseases.

## Figures and Tables

**Table 2:** Bacterial metabolites and their effects in periodontal pocket.

Bacterial Metabolites	Effects on Periodontal Pocket
Acetic, propionic and butyric acid [53]	Inhibition of T and B lymphocytes proliferation; Inhibition of IL-2, IL-4, IL-5, IL-6, and IL-10 production [63]
H_2_S [53,64]	Secretion of IL-1β and IL-18; apoptosis in gingival fibroblasts, periodontal ligament cells, and lymphocytes; impaired collagen synthesis [53,64]

## Data Availability

No new data were created or analyzed in this study.

## Abbreviations

The following abbreviations are used in this manuscript:

SCFAs	Short-chain fatty acids
IBD	Inflammatory Bowel Disease
PSD	Polymicrobial Synergy and Dysbiosis
WMS	Whole Metagenome Shotgun sequencing
LPS	Lipopolysaccharide
OMV	Outer Membrane e Vesicles
IL-1β	Interleukin-1β
IL-1	Interleukin-1
IL-6	Interleukin-6
TNF-α	Tumor Necrosis Factor-α
Kgp	lysine-specific gingipains
LtxA)	leukotoxin
CDT	Cytolethal Distending Toxin
GCF	Gingival Crevicular Fluid
H_2_S	Hydrogen Sulfide
NAFLD	Non-Alcoholic Fatty Liver Disease
Ocln	Occludin
CRC	Colorectal Cancer
NGS	Next-Generation Sequencing
CD	Crohn’s disease
GC	Gastric Cancer
ChG	Chronic Gastritis
GIT	Gastrointestinal Tract
MSSM	Micro-scale Spatial Metagenomics
MALDI-MSI	Matrix-Assisted Laser Desorption/Ionization Mass Spectrometry Imaging
DESI-MSI	Desorption Electrospray Ionization Mass Spectrometry Imaging
NSPT	Non-surgical periodontal therapy
SPC	Supportive Periodontal Care
NSPI	Non-Surgical Peri-implant submarginal instrumentation
MINST	Minimally Invasive Non-Surgical Therapy
